# Fenofibrate Nanocrystals Embedded in Oral Strip-Films for Bioavailability Enhancement

**DOI:** 10.3390/bioengineering5010016

**Published:** 2018-02-13

**Authors:** Bhavesh D. Kevadiya, Manish Barvaliya, Lu Zhang, Ashish Anovadiya, Harshad Brahmbhatt, Parimal Paul, Chandrabhanu Tripathi

**Affiliations:** 1 Department of Chemical, Biological and Pharmaceutical Engineering, New Jersey Institute of Technology, Newark, NJ 07102, USA; lz74@njit.edu; 2 Department of Pharmacology, Government Medical College, Bhavnagar 364002, Gujarat, India; drmanishbarvaliya@gmail.com (M.B.); ashishanovadiya@yahoo.com (A.A.); cbtripathi@yahoo.co.in (C.T.); 3 Analytical Discipline and Centralized Instrument Facility, The Academy of Scientific & Innovative Research (AcSIR), Central Salt and Marine Chemicals Research Institute, Council of Scientific and Industrial Research (CSIR), G.B. Marg, Bhavnagar 364002, Gujarat, India; harshadb@csmcri.org (H.B.); ppaul@csmcri.org (P.P.)

**Keywords:** bioavailability, fenofibrate, media milling, pharmacokinetics, UV imaging

## Abstract

The aim of the present study was to make a fenofibrate (FNB) nanocrystal (NC) by wet media milling, characterizations and formulates into oral strip-films (OSFs). Mechanical properties, redispersion study, and solid-state characterizations results suggested that reduction of drug crystal size at nanoscale and incorporation into OSFs does not affect the solid-state properties of the drug. *In vitro* dissolution kinetics showed enhanced dissolution rate was easily manipulated by changing the thickness of the OSF. *In situ* UV-imaging was used to monitor drug dissolution qualitatively and quantitatively in real time. Results confirm that the intrinsic dissolution rates and surface drug concentration measured with this device were in agreement with the USP-IV dissolution profiles. *In vivo* pharmacokinetics in rabbits showed a significant difference in the pharmacokinetics parameter (1.4 fold increase bioavailability) of FNB NC-loaded OSFs as compared to the marketed formulation “Tricor” and as-received (pristine) drug. This approach of drug nanocrystallization and incorporation into OSFs may have significant applications in cost-effective tools for bioavailability enhancement of FNB.

## 1. Introduction

Fenofibrate (FNB) is a potent and well-known cholesterol-reducing agent, a pro-drug of fenofibric acid with neutral charge, lipophilic nature, and a very low water solubility (<0.5 mg/L with logP = 5.2), and is widely applied clinically to treat LDL-C and high triglyceride (TG) levels [1,2,3,4,5,6]. It acts by stimulating the activity of peroxisome proliferator-activated receptor-α (PPAR-α), a member of the PPAR subfamily of nuclear receptors that modulate the transcription of genes that regulate lipid metabolism [1,2]. Activation of PPAR-α increases expression of lipoprotein lipase and APO-AI (Apolipoprotein A1), which facilitates removal of TGs from VLDL and the reverse cholesterol transport to HDL, respectively. According to the Biopharmaceutics Classification System, FNB is a class II drug with low water solubility, and dissolution rate of FNB may limit its bioavailability (only 30% in humans) in the gastrointestinal (GI) tract [6,7]. However, many different formulations of FNB have been investigated to improve its overall solubility in order to enhance its bioavailability, such as silicate and mesoporous silica [4,7,8,9], micro emulsifying and micronized formulations [1,10,11], and liposomes [6]. The complexity associated with these materials due to their 3D structure involves careful design and engineering, rendering them unsuitable for these types of molecules and possible toxicity-related issues [12,13,14,15].

In the past decades, nanosuspension production using particle engineering technology as a drug delivery system has been an emerging strategy for enhancing bioavailability of FNB in the pharmaceutical industry [16,17,18,19,20,21]. Aqueous suspensions of nanocrystals (NCs) are carrier-free, colloidal dispersions of pristine drug particles in water with a mean particle size in the nanometer range, typically between 10 nm and 1000 nm. Despite progress in this field, while NCs deliver exceptional pharmaceutical features, they also render them thermodynamically unstable and encourage agglomeration, creaming, and crystal growth of smaller particles in suspension by changing in the Gibbs free energy (ΔG) (Ostwald ripening) [20,22,23,24,25]. Besides, suspensions are inconvenient to pack and transport, which severely restricts direct oral application of NCs [26]. Furthermore, the transport of NCs across the GI epithelium is practically challenging due to the low drug NC binding affinity to the GI epithelium. The low cellular transport of NCs results in an inadequate oral bioavailability [27]. Hence, it is crucial to convert NC suspensions into a dry solid oral dosage form without deterioration of the physical stability and plasma drug concentrations from the therapeutic window. The drying of NC suspensions can be achieved by various methods such as fluidized bed drying, spray drying, freeze-drying, and hydrogels [24,28,29]. However, these drying techniques are frequently associated with permanent aggregation or drug particles, which negatively affects NC recovery from the solid dosage form, leading to reduced oral bioavailability of drugs [30]. Thus, it is a big task to develop a solid dosage form that has a good oral bioavailability of FNB.

Oral drug delivery is a major challenge in the field of pharmaceutical research due to pH variations and proteolytic enzymes, which are present in GI track [31]. Oral strip-films (OSFs) containing drug NCs are novel solid dosage forms that disintegrate within a very short period of time when placed in the mouth without water intake or mastication [16,30,32,33]. OSFs containing NCs have numerous benefits over conventional dosage forms. They are of significance during emergency circumstances whenever an immediate onset of action is desired [34] and stress-free for patients who have difficulty swallowing, such as the elderly, pediatric patients, and others suffering from oral pain due to mucositis or after oral surgical treatment, or those with nausea [30]. Moreover, OSFs also promise more accurate dosing of drugs compared to gels and ointments. Indeed, OSFs seem to be a great substitute for the drug NC solidification process without agglomeration and converts suspended drug NCs into a solid dosage form for patients in all age groups.

**Figure 1:**
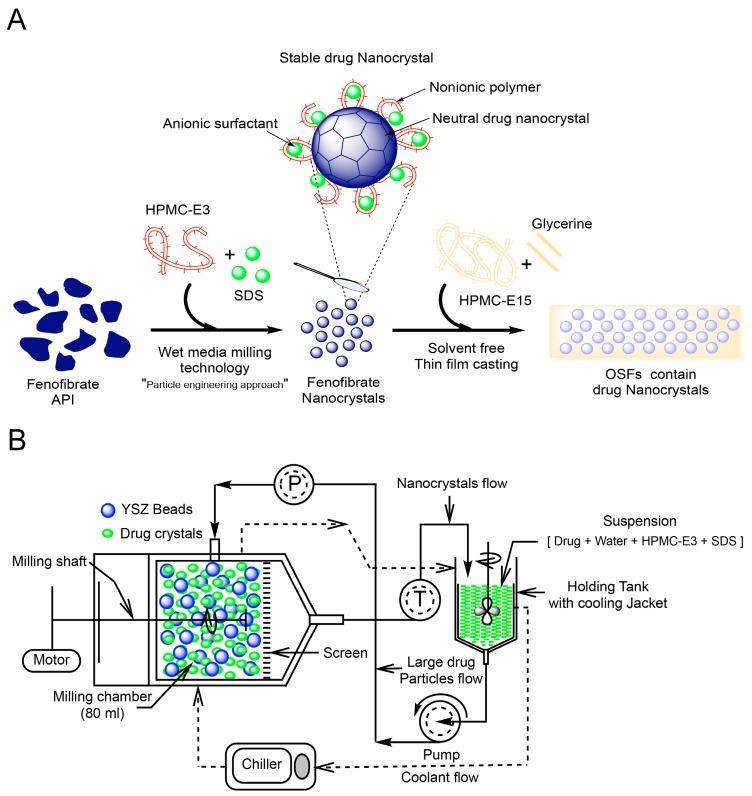
Graphical representation of preparations of OSFs (oral strip-films) containing FNB-NCs (fenofibrate nanocrystals), Here, API: Active Pharmaceutical Ingredient; HPMC-E3/HPMC-E15: Hydroxypropyl Methylcellulose-E3/E15; SDS: Sodium Dodecyl Sulfate; YSZ: Yttria stabilized zirconia) (**A**) and Schematic diagram of the nanomilling process operating in recirculation mode (P; Pressure and T; Thermocouple, green balls represent SDS micelles) (**B**).

Toward achieving this objective, FNB-NCs were prepared by wet stirred media milling (WSMM) and then transformed into OSFs containing drug NCs by using a solvent-free method with tape casting equipment. The solid state of OSFs was investigated by means of XRD analyses, FTIR, Raman spectroscopy, TGA-DTA, and DSC. The influence of thickness on mechanical properties of the film was investigated. Laser diffraction particle size analysis was used for the FNB-NCs particle size distribution and re-dispersed from OSFs. SEM observed the morphology of the structure of the films. The drug content for films containing NCs with different thicknesses was studied. *In vitro* drug dissolution experiments were performed using an automated USP-IV. The drug release kinetics was elucidated via different mathematical models. Moreover, *in situ* dissolution behavior of OSFs containing drug NCs was characterized by a surface dissolution imaging system (SDI), which allows for monitoring temporal variation of dissolved FNB concentration. *In vivo* pharmacokinetics (PK) of formulated FNB-NCs in rabbits was examined. The overview of entire process objective of work was illustrated in Figure 1A.

## 2. Materials and Methods

### 2.1. Materials

For the present study, fenofibrate (FNB) was obtained from Jay Radhe Sales, Ahmedabad, India. Sodium dodecyl sulfate (SDS) was purchased from GFS Chemicals Inc., Columbus, OH, USA. Hydroxypropyl methyl cellulose (HPMC) (METHOCEL E15 Premium LV, Mw = ~40,000 and Methocel E3 Premium LV, Mw = ~20,300) was purchased from Dow Chemical Company, Midland, MI, USA. Glycerin was purchased from Sigma–Aldrich, St. Louis, MO, USA. All chemicals were of analytical grade.

### 2.2. Preparation of Coarse Drug Suspensions

The coarse drug suspensions were prepared by dispersing of SDS (0.075 g)and HPMC-E3 (1.0 g) dry powered in deionized water and mixed for 60 min at 300 rpm by shear mixer (Fisher Scientific Laboratory Stirrer, Pittsburgh, PA, USA) to ensure proper HPMC-E3 and SDS micelles forms [35]. 10 g of FNB powder was weighed and added to HPMC-E3-SDS solution to obtain a homogenous coarse suspension.

### 2.3. Preparation of Drug Nanocrystals by Wet Stirred Media Milling (WSMM)

Coarse drug suspensions prepared as mentioned above were subsequently milled in a Netzsch wet media mill (Microcer, Fine particle technology LLC, Exton, PA, USA). Yttrium-stabilized zirconium (YSZ) beads with a nominal size of 400 μm were used as the milling media and a 200-μm screen was used to hold the beads in the milling chamber. YSZ beads with a 50 mL bulk volume were loaded to the milling chamber with 80 mL volume capacity. The milling process was performed with the coarse drug suspension fed at a rate of 120 mL/min using a peristaltic pump. The operating parameters for mill are as follows: 3200 rpm milling speed, 2.0 h running time, and 11.8 m/s tip speed. The processing temperature inside the mill was kept up at less than 32 °C by controlled circulation of coolant through the outer jacket by a chiller (Advantage Engineering, Inc., Greenwood, IN, USA). The milling process was performed in recirculation mode. In this technique, the suspension circulates from the holding tank through the milling chamber, exits through the screen, and returns back to the holding tank. Figure 1B shows a schematic of the WSMM process used for NC suspensions preparation. The obtained FNB-NCs were directly incorporated into OSFs by mixing with HPMC-E15.

### 2.4. Preparation of OSFs Containing NCs

OSFs containing NCs were prepared by a modified solvent-free casting technique by using tape-casting equipment (Model TC-71LC, HED International Inc., Ringoes, NJ, USA) [36]. Briefly stated, an 8% HPMC-E15 polymer solution was prepared by adding a weighed quantity of HPMC-E15LV and 5% glycerin to the water (on *w*/*w* basis) at 90 °C (Dow Chemical Company protocol). The hot polymer solution was allowed to cool down to room temperature while being stirred continuously. This method was selected based on previous studies, taking into account the final suspension viscosity and film quality [16,32]. FNB-NC suspension was then added to the HPMC-E15 solution in a 1:2 (*w*/*w*) ratio and mixed using a standard impeller (VWR VOS 16 Overhead Stirrer, VWR International, Radnor, PI, USA) stirring at 300 rpm until the film-forming suspension was completely homogeneous. The mixture was then placed in a planetary centrifugal mixer (Thinky Mixer ARE-310, Thinky, Inc., Laguna Hills, CA, USA) and subjected to 5 min of mixing followed by 5 min of defoaming to remove bubbles at speed 1500 rpm. The mixed suspension was cast on fluoropolymer coated polyester substrate and thickness including in Table S1 [36] (Scotchpak 9744, 3M Drug Delivery Systems, St Paul, MN, USA) using a doctor blade film applicator (Elcometer-3700, Elcometer Instruments, Rochester Hills, MI, USA), then dried at 45 °C for 30 min (Model TC-71LC, HED International Inc., Ringoes, NJ, USA). To better investigate the drug NC solid state, a placebo formulation with the equivalent composition, including the glycerin, but without drug NCs was also prepared as a control. OSFs were packed in individual airtight polyethylene seal packs immediately after preparation and stored at RT.

### 2.5. Particle Size and Zeta Potential Analysis

The particle size was analyzed by a Coulter Beckman LS-13-320 (Beckman Coulter, Miami, FL, USA). Refractive index values of 1.55 for the FNB particles [37] and 1.33 for the measurement medium were used. Prior to the size measurement, milled drug NC suspension samples (~2 mL) were diluted in a vial with 15 mL of HPMC-E3-SDS solution using a vortex mixer (Fisher Scientific Digital Vortex Mixer, Hampton, NH, USA), which rotated at 1500 rpm for 1 min. The re-dispersion particle size testing was conducted to evaluate the recovery of the drug NCs from OSFs in an aqueous system and implemented as follows: circular OSFs with an area of 0.712 cm^2^ from each formulation were put in 15 mL of de-ionized water for OSF disintegration. NC particle size was measured after 10 min of magnetic stirring followed by 1 min vortex mixing at 1500 rpm [16]. The zeta potential of NC suspensions were measured by the Delsa™ Nano Zeta Potential Analyzer (Beckman Coulter, Miami, FL, USA).

### 2.6. Analytical Characterization

All OSF samples were characterized by powder X-ray diffraction (XRD) (Philips PW3040 X-ray Diffractometer, Siemens, Boston, MA, USA) using a Philips X’celerator detector and Philips X’Pert Data Collector with a curved Ni-filtered Cu-Kα (λ = 1.542 Å) radiation and a scanning rate of 2°/min over a 2θ range of 10–40°. Attenuated total reflection Fourier transform infrared (ATR-FTIR) spectra of the samples were generated by a Magna Model 560 instrument (Nicolet Instrument Corporation, Madison, WI, USA) attached to an attenuated total reflectance (ATR) accessory with a single reflection Zn-Se crystal (MIRa-cle; Pike Technologies, Madison, WI, USA). FT-Raman shift was measured with an EZ Raman system from Enwave Optronics Inc., Suite A Irvine, CA, USA. This system was equipped with an HRP 8 high throughput fiber probe coupled to a MicroView adapter with a 10× objective 10 μm spot size, and EZ Raman Reader software-I was used for data acquisition. Thermogravimetric analysis (TGA) was carried out within 30–250 °C at the heating rate of 10 °C/min under nitrogen flow (20 mL/min) using a TGA/DSC STARe system (Mettler-Toledo, Inc., Toledo, OH, USA). Differential scanning calorimetry (DSC) studies were carried out in the range of 30–150 °C at the rate 10 °C/min under nitrogen flow (20 mL/min) using a Polymer DSC, TGA/DSC STARe system (Mettler-Toledo, Inc., Toledo, OH, USA). The surface morphology of NCs and OSFs was examined via scanning electron microscope (SEM; LEO 1530 VP, Carl Zeiss NTS Ltd., Cambridge, UK). All OSF and NC samples were pre-coated with carbon using a sputter coater to enhance conductivity.

### 2.7. Properties of OSFs

#### 2.7.1. OSF Thickness Measurement

Ten circular samples ~0.712 cm^2^ in area were punched out of each film from random locations across the OSF. The thickness of the circular OSF samples was measured using a calibrated digital Micrometer screw with an accuracy of 0.001 mm (Mitutoyo, Aurora, IL, USA) and the mean value was reported.

#### 2.7.2. Tensile Properties

Tensile testing was conducted using a mechanical testing machine equipped with a TA-XT plus Texture Analyzer (Stable Microsystems, Surrey, UK). The OSF was cut into 50 mm × 15 mm strips and tensile tests were done according to the procedure reported in a previous work [16,32]. The strips were held between two clamps and pulled by the clamps at the rate of 1 mm/s to the breaking point (i.e., tensile failure). Measurements of the tensile properties of the OSFs were done in triplicate for each formulation, and tensile strength (TS), yield strength (YS), elongation at break (E) and Young’s modulus (YM) were calculated [16].

#### 2.7.3. Determination of Drug Content

The drug content in the OSFs was determined using a UV absorbance wavelength of 290 nm using previously constructed calibration curves. Ten circular samples ~0.712 cm^2^ in area were punched out of each film from random locations across the OSF and dissolved in 250 mL SDS medium (7.2 mg/mL, pH = 8.1) for a period of 24 h at 25 °C. The drug concentration was then calculated using the average of the ten OSF samples.

### 2.8. In Vitro Flow-Through Cell Drug Dissolution System

*In vitro* drug dissolution from OSFs was investigated using an automated flow-through cell dissolution method (USP-IV; Sotax CE7 smart, Sotax, Basel, Switzerland) [16,32]. Auto quantification of sample UV absorbance was performed by Win-Sotax software (Version 2.6.4, Basel, Switzerland). The equipment was fitted with 22.6 mm cells (internal diameter). Dissolution studies were carried out using SDS (7.2 mg/mL, pH = 8.1) as release media at 37 ± 0.5 °C. A ruby ball (Ø 5 mm) and 3 g of glass beads (Ø 1 mm) were placed at the bottom of the cone to ensure laminar flow of fluid entering the cell. The circular OSF samples (0.712 cm^2^ in area) were placed on the top of the glass beads and covered with 2 g of glass beads (Ø 1 mm), which prevented the OSF from floating in the flowing media. Undissolved drug particles were kept in the vessel by a membrane disc filter, pore size 0.2 µm, 25 mm diameter (Supor^®^-200, Pall Life Science, Port Washington, NY, USA) placed at the top of the cyclone cone. The flow rate was maintained at 16 mL/min. All experiments were performed at least four times and the average of drug dissolution data was plotted as a function of time.

### 2.9. UV Imaging Release Experiments

#### 2.9.1. Surface Dissolution Imager Setup

Mechanisms of *in vitro* drug dissolution from OSFs were further elucidated using a Sirius Surface Dissolution Imager (SDI) UV imaging system (Sirius Analytical Instruments Ltd., East Sussex, UK) and an ActiPix™ D100 UV Area Imaging System (Paraytec Limited, York, UK) employing an Actipix flow-through-type dissolution method with a detection area of 9 mm × 7 mm (1280 × 1024 pixels). Images were recorded at a rate of 3.82 frames per second (sub sampling of every 10 min was applied) and the data were analyzed with Actipix D100 software version 1.2 (Paraytec Ltd., York, UK) with 10 × 1 horizontal pixel binning (Pixels = 7 μm × 7 μm). A pulsed xenon lamp was used as the light source and imaging was performed at 280 nm single wavelength filter for detection (band width 10 nm). The quartz dissolution cell with 7.5 mm × 3.0 mm (light path length) × 63 mm (H × W × L) contained approximately 500 µL dissolution media with the inserts in place. The calibration curve of drug in surface dissolution Imager was presented in Supplementary Materials Figure S1.

#### 2.9.2. *In Situ* Real Time UV Image Monitoring

Dark images (lamp turned off: 10 s) and reference images (10 s) were recorded with the quartz cell filled with release media (SDS, 7.2 mg/mL, pH = 8.1) and an empty stainless steel cylinder positioned in the sample compact holder. Data collection was then started and allowed to proceed for 60 s. An OSF sample was manually set on the surface of the stainless steel sample cup (Supplementary Materials Figure S2). First, inert material was inserted from the bottom of the mold as supportive material in order to facilitate the compaction of the film sample. Then, the OSF sample was placed on the upper part of the inert material to create a flat surface to avoid flow effect of dissolution media on the OSF sample during analysis. Then the sample-containing steel cylinder was inserted in the 3D printed holder (Supplementary Materials Figure S2A,B). After the insertion of the OSF sample, the quartz cell’s cavity was filled with dissolution media at a constant flow rate of 100 µL/min. Measurements were taken for 1 h with at least three repeats for each OSF at 37 ± 0.5 °C. Downstream of the sample, at a programmed measurement area of the pixilated detector, the UV absorbance at 280 nm was measured using the instrumental UV area imaging software(Actipix D100 software version 1.2, Paraytec Ltd., York, UK). By measuring the absorbance over time in combination with the flow rate, the software estimated the surface concentration and intrinsic dissolution rate (IDR) of the drug sample.

### 2.10. Pharmacokinetic Study

New Zealand white rabbits of either gender (48–52 weeks old, 1.5–1.8 kg, obtained from Central Animal House, Government Medical College, Bhavnagar, India, Reg. No. 577/GO/c/02/CPCSEA) were used in the pharmacokinetic study (six rabbits per group). The animals were maintained at the animal holding room at the Department of Pharmacology, Government Medical College, Bhavnagar, India. Pharmacokinetic (PK) studies were performed after approval from the Institutional Animal Ethics Committee (IAEC), Government Medical College Bhavnagar, Gujarat, India [IAEC No. 42/2015]. The animals were kept in a 12 h light-dark cycle under controlled room temperature (25 ± 2 °C) with relative humidity 50–60%. The animals were given standard laboratory diet and allowed to acclimatize for at least one week before starting the experiments. Animal caring, handling, and study procedures were performed as per the guidelines issued by the Committee for the Purpose of Control and Supervision of Experiments on Animals (CPCSEA), Ministry of Environment and Forest, Government of India. Each animal was fasted for 12 h prior to the studies and water was made available ad libitum. All the formulations were administered orally/buccal administration at an equivalent dose of 30 mg/kg body weights of FNB. Eighteen rabbits were randomly allocated into three groups of six animals each. The first group of animals received oral Tricor (suspension), while the second group of animals received pristine FNB (suspension), and the third group received OSFs (5.28 mg/cm^2^ of FNB-NC-D). Blood (approximately 0.7 mL) were sampled from the marginal ear vein at the following predetermine time: 0 (pre-dose) and 0.5, 1, 1.5, 2, 4, 6, 8, 12, 24 and 48 h after administration. Plasma stored at −20° until HPLC analysis. The pharmacokinetic parameters assayed were by calculations of (a) total area under the curve (AUC)_0–∞_, (b) mean residence time (MRT), (c) peak plasma concentration (C_max_), and (d) time to reach the maximum plasma concentration (T_max_).The quantification of FNB in plasma was done using a validated RP-HPLC method. The FNB was extracted in methanol from plasma samples, and analyzed by an HPLC system consisting of a photodiode array detector (Shimadzu Prominence Model) and a reverse-phase C18 column (Enable C18 column, Particle size; 150 mm × 4 mm × 5 µM). The mixture of acetonitrile and water (70:30, *v*/*v*) with 0.1% phosphoric acid was used as the mobile phase for analysis. The injection volume was 50 µL, retention time of FNB was 4.6 min, and detection wavelength for FNB was 280 nm.

## 3. Results and Discussion

### 3.1. Particle Size and Zeta Potential Effect on Stability of NCs

**Figure 2:**
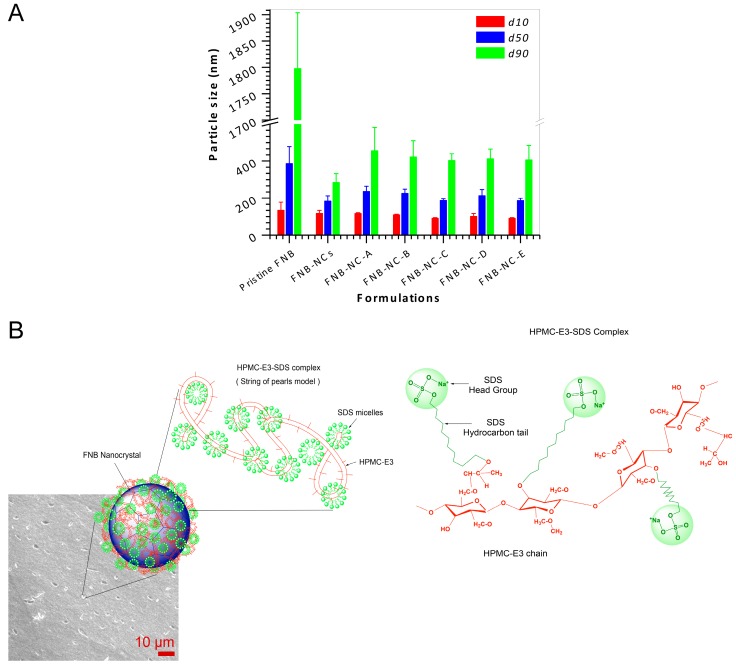
Drug nanocrystal recovery from OSFs by redispersion using deionized water (**A**) and the string of pearls model with schematic representation of the supramolecular structure of HPMC-E3-SDS complex stabilization of drug nanocrystals in the OSFs (**B**).

Using HPMC-E3 and SDS as stabilizers during WSMM produced stable FNB-NCs. After 2 h of milling, FNB particle size range was reduced from of 1798 (before milling) nm to 285 nm, as shown in Figure 2A. The drug NCs were re-dispersed in distilled water from OSFs. After FNB-NC re-dispersion from OSFs in de-ionized water, the particle size of FNB-NCs was 420/210/102 nm (d90/d50/d10). Figure 2A shows that there was only a slight increase in NC particle size after re-dispersion from OSFs compared with that of the FNB-NC suspension. This can be attributed to the fact that FNB-NCs strongly attach to the HPMC polymer chain due to the HPMC-SDS stabilizer adsorbed onto the surface of the FNB-NCs during milling. Zeta potential provides certain evidence about the surface charge properties and long-term physical stability of NC suspensions. For a physically stable NC suspension exclusively stabilized by electrostatic repulsion, a zeta potential of ±30 mV is essential. In the case of combined electrostatic and steric stabilization, roughly ±20 mV is adequate [38]. The zeta potential of nanosuspensions was −22.3 ± 1.7 mV, which provided assurance of combined electrostatic and steric stabilization.

Apart from the zeta potential value, polymer and surfactant types also play a significant role in FNB-NC suspension stability and interaction with the biological system. Polymers with charge are more rapidly cleared from circulation than nonionic polymers. Neutral and anionic polymers are less toxic than cationic polymers [39]. A surfactant-stabilized NC surface reduces interfacial tension by allowing attractive water–surfactant interactions, stabilizing the crystal surfaces and reducing tendency for NC agglomeration [25]. The HPMC-E3 with SDS is an appropriate combination of stabilizers due to their semi-flexible hydrophobic chains, which have an affinity for the hydrophobic surface of the FNB-NCs. According to the particle size and zeta potential measurements, the HPMC-E3-SDS complex was a sufficient combination to stabilize the drug NCs suspension.

Based on previous published reports [39,40,41,42], the microstructure of HPMC-E3-SDS complexes formed likely followed a “String of pearls model”, as shown in Figure 2B. Typically, the HPMC-E3 functions as a nucleation point for SDS micellization along the HPMC-E3 chain backbone with one micelle of SDS having one or more HPMC-E3 chains involved in to it. Therefore, HPMC-E3 polymer chains thread-string either penetrates through the SDS micelles or is located at the outer hydrophilic shell of the SDS micelles. In terms of molecular recognition, the charged head groups of SDS (–OSO3Na^+^) and the gap between these charged head groups on a micellar surface where the long hydrocarbon chains of SDS are exposed to water offer two different interaction sites for intermolecular attraction, which permits the micelle to identify different segments of the HPMC-E3 chain (Figure 2B).

### 3.2. Solid-State Characterization

#### 3.2.1. X-ray Diffractograms

**Figure 3:**
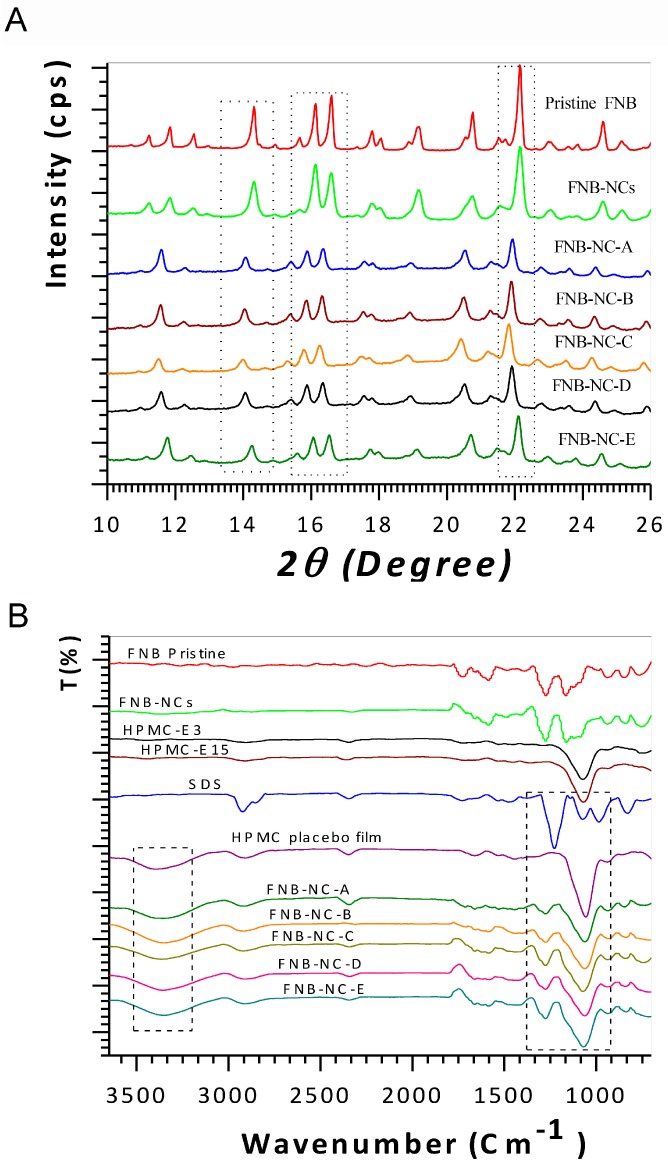
X-ray powder diffraction (XRD) patterns (**A**) and Fourier transform infrared spectroscopy (FTIR) analysis (**B**).

X-ray diffraction (XRD) could be used to investigate potential alterations in the internal structure of drug NCs. Figure 3A shows the XRD spectra of pristine FNB, FNB-NCs, and FNB-NCs incorporated into OSFs. As seen from Figure 3A, the pristine FNB exhibits characteristic peaks at diffraction angles 2θ of 11.8°, 14.3°, 16.10°, 16.60°, 17.75°, 19.20°, 20.80°, 22.10° and 24.60°. The FNB-NCs and all OSFs also showed similar peaks, but with slightly lower peak intensities were attributed to masking effect of polymers.

#### 3.2.2. ATR-FTIR and FT-Raman Spectroscopy Analysis

The ATR-FTIR spectra for the pristine FNB, FNB-NCs, and OSFs containing FNB-NCs are shown in Figure 3B. The spectra of pristine FNB and FNB-NCs revealed the following characteristic absorption bands: 1722 cm^−1^, which corresponds to –OH stretching, 1580 cm^−1^, which corresponds to –C=C or –CH3 stretching–bending, 1270 cm^−1^, which corresponds to –C=O stretching and –OH bending, and 1162 cm^−1^, which corresponds to –C–O–C ring stretching. Moreover, the characteristic peak of HPMC at 1067 cm^−1^ is attributed to –C–O–C stretching of the polysaccharide groups. A wide peak at 3387 cm^−1^ represents the –OH intermolecular hydrogen bonding and the triple peak between 930 cm^−1^ and 1270 cm^−1^ in all OSF formulations corresponds to the –C–O– groups of HPMC. The peak at 2900 cm^−1^ is due to stretching of the methyl and propyl groups of HPMC [43,44,45]. The peaks at 1075 cm^−1^ and 1226 cm^−1^ show symmetric vibrational and asymmetric vibrational stretching of –SO_2_ head groups of SDS, respectively. The peak at 2920 cm^−1^ is due to –C–H stretching of SDS [46]. Compared to the peaks of carrier materials, (Formulations A–E), little shifting in the peaks at 1580–1722 cm^−1^ and 1661 cm^−1^ was observed in OSFs containing FNB-NCs. Slight shifting of SDS peaks from 1920 cm^−1^ to 1917 cm^−1^ and from 1226 cm^−1^ to 1270 cm^−1^ was also observed. Characteristic peaks of HPMC shifted from 3387 cm^−1^ to 3358 cm^−1^ and from 1067 cm^−1^ to 1056 cm^−1^, indicating that the HPMC-E3-SDS complex were formed the inclusion of the FNB-NCs inside the HPMC polymeric matrix.

Comparisons of the FT-Raman spectra of pristine FNB and FNB-NCs incorporated in OSFs are shown in (Supplementary Figure S3). Sharp bands and individual separated peaks can be observed throughout the FT-Raman spectra of crystalline forms of FNB-NCs. For example, two distinct peaks were observed in the characteristic carbonyl-stretching region between 1600 cm^−1^ and 1655 cm^−1^ [47]. Together, these ATR-FTIR and FT-Raman spectroscopy studies suggest that interactions occur between stabilized FNB-NCs and HPMC, and that particles remained in the crystalline state after the WSMM process.

#### 3.2.3. Thermal Analysis

**Figure 4:**
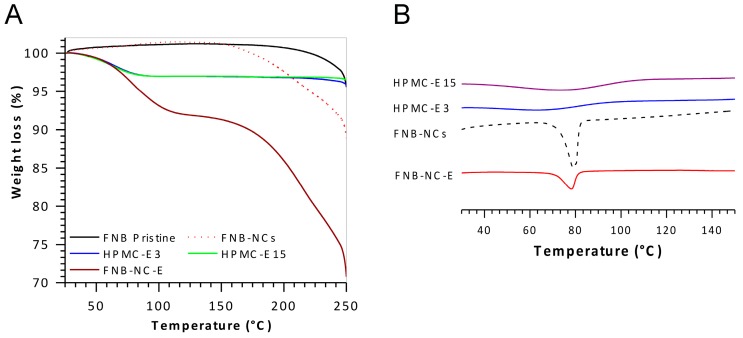
Thermogravimetric analysis (TGA) (**A**) and Differential scanning calorimetry (DSC) patterns (**B**).

The TGA curves of the pristine FNB, FNB-NCs, and OSFs containing FNB-NCs are shown in Figure 4A. The TGA curve of pristine FNB shows a single step weight loss at ~248 °C. This strong endothermic peak, also observed in TGA patterns, was an indication of FNB degradation. The TGA curve of FNB-NCs demonstrated endothermic weight loss peaks in four steps at 65 °C, 120 °C, 200 °C and 248 °C. The first weight loss peak observed for HPMC-E3 corresponds to the evaporation of water. Curves of OSFs containing FNB-NCs clearly showed weight loss in three different steps at 80 °C, 215 °C and 248 °C. These steps were directly correlated with those of the HPMC placebo OSF samples (85 °C, 200 °C and 235 °C) and FNB-NCs (248 °C).

The DSC thermograms are shown in Figure 4B. The FNB-NCs showed a sharp endothermic peak near 79.25 °C, which is near to the ideal melting temperature of FNB [48]. The melting endotherms of both HPMC-E15 and HPMC-E3 were shown to be between 65 °C and 70 °C (water contents). The OSFs containing FNB-NCs exhibited a melting endotherm at ~80 °C. Comparing the curves of FNB-NCs and OSFs containing FNB-NCs, peak intensity is marginally lower for the OSFs (FNB-NC-E) suggesting that some amorphous FNB might have been formed during the process and incorporations.

### 3.3. Morphological Characterization

**Figure 5:**
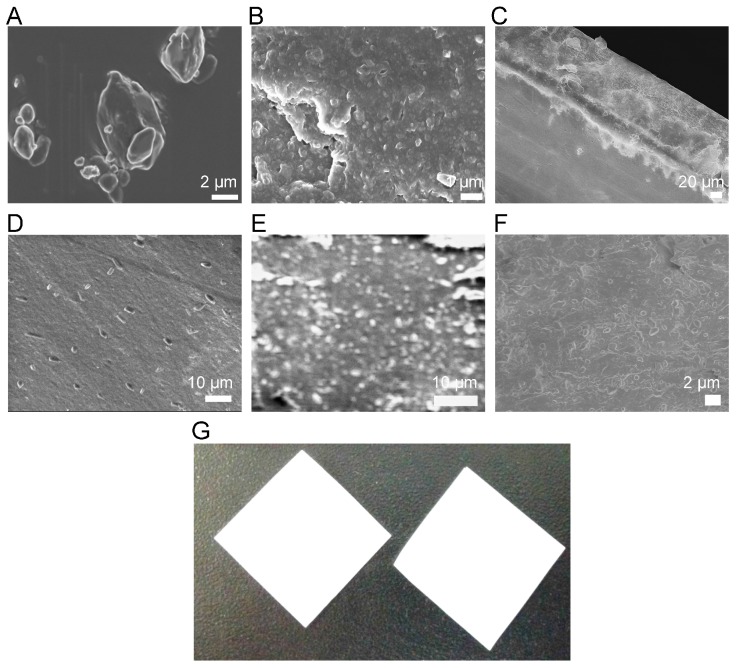
The SEM picture of a pristine FNB particle (**A**), FNB-NC suspension (**B**), top cross section view of OSF (**C**), surface view of OSF (**D**), magnified cross section view of OSF (**E**), magnified top surface OSF view (**F**) and Picture of OSFs containing drug NCs (2 cm × 2 cm) (**G**).

SEM was employed to investigate the surface morphology of the samples. SEM images of pristine FNB, FNB-NCs, and OSFs containing FNB-NCs are shown in Figure 5. The pristine FNB (Figure 5A) had an irregular shape with rough surface morphology particle. After WSMM, the large FNB crystals were broken into NCs in the presence of the HPMC-E3-SDS stabilizer complex (Figure 5B), smoothing the angular surfaces of the FNB particles. FNB-NCs had a crystalline and probable granular nature with clearly separated particles, while well-dispersed FNB-NCs with slight adhesion to the HPMC polymeric matrix were observed in OSFs (Figure 5C–F). Compared with the particle size of coarse suspensions of FNB (d90 = ~1.80 µm, Figure 2A), the particle size of FNB-NCs and FNB-NCs in the OSFs was significantly reduced to ~250–450 nm, (Figure 5C–F).

### 3.4. Properties of OSFs

#### 3.4.1. Drug Content and Thickness

Visual examination showed that the prepared OSFs were opaque-white (due to polymer-drug mixture) and uniform without any cracks (Figure 5G). The OSFs were easily handled and, as unit solid dosage forms containing drug NCs, they were simply prepared by cutting sheets into appropriate sizes and shapes (Table 1). Dry film thickness was modulated by changing the wet thickness using a doctor blade and ranged from 45.9 ± 2.7 µm to 165.0 ± 5.5 µm for film samples FNB-NS-A through FNB-NS-E with increasing thickness. The prepared OSFs showed good thickness uniformity.

To evaluate drug content and uniformity of films, content uniformity tests were performed on all film formulations to evaluate thickness, mass of drug per unit area, and drug loading by wt. % (Table 1). OSFs were found to be adaptable and free from budding. Drug content remained (between 31.43 ± 1.5% and 37.07 ± 1.4%) with increased thickness of films.

#### 3.4.2. Mechanical Properties of OSFs

Mechanical properties of OSFs were assessment by measuring tensile strength (TS), yield strength (YS), Young’s Modulus (YM), and elongation at break (E) (Table S1). The TS and E has been increased with OSFs thickness compared to thin OSFs. The mechanical strength of the films higher in order FNB-NC-E > FNB-NC-D > FNB-NC-C > FNB-NC-B > FNB-NC-A. The FNB-NC-E was shown higher mechanical strength due to excessive amount of HPMC in formulations.

### 3.5. In Vitro Dissolution Studies

**Figure 6:**
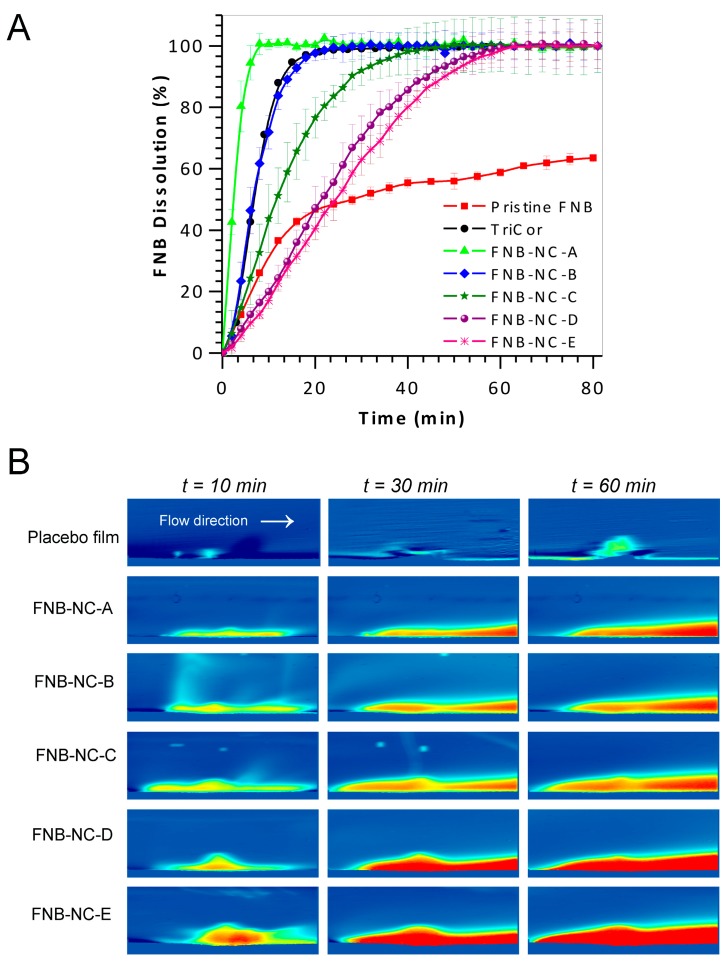
*In vitro* drug dissolution profiles of FNB in sodium dodecyl sulfate (pH 8.1) at 37 ± 0.5 °C; data represent mean ± SD (*n* = 4) (**A**) and Selective snapshots of real-time UV imaging of drug release and dissolution of OSFs under flow conditions (100 µL/min) in SDS media, pH 8.1 (7.2 mg/mL) at 280 nm. UV absorbance images obtained after 10 min, 30 min and 60 min (**B**).

*In vitro* drug dissolution studies were carried out at pH 8.1 (SDS conc. = 7.2 mg/mL). As demonstrated in Figure 6A, the dissolution rate of OSFs containing FNB-NCs was clearly higher than that of pristine FNB and easily manipulated by varying the thickness of the OSFs, as thicker films exhibited slower dissolution. Approximately 100% of FNB was released from the OSF with thickness 45.90 ± 2.7 µm (FNB-NS-A) within 8 min, although the same 100% release was reached at longer times for thicker films: 22 min (84.5 ± 8.0 µm), >42 min (115.8 ± 4.7 µm), >55 min (151.2 ± 5.7 µm), and >63 min (165.0 ± 5.5 µm). This indicates that reduced dissolution velocity from thicker OSFs might be due to the longer path length across for NCs in the HPMC polymeric matrix. Moreover, drug NC dissolution rate was generally faster than non-milled drug particles. Significant increased surface area and decreased particle size in nanometer scale can lead to increased drug dissolution rate and better mucoadhesion of film, which can decrease mucosal epithelial transport time and lead to increased oral bioavailability [16,23,28,29,32,35,37].

### 3.6. In Situ Real-Time Drug Dissolution Analysis

*In situ* real-time dissolution of OSFs containing FNB-NCs was studied under continuous flow conditions. In order to shown real-time drug dissolution analysis, these OSFs were mounted on a 2 mm in diameter cylinder and inserted in the UV imager. The plane perpendicular to the surface of the OSFs was available for imaging. The ActiPix SDI 300 UV software version 1.2 (Paraytec Ltd., York, UK) was used calculation of the IDR from a three-dimensional downstream cavity of the quartz sample cell by UV absorbance for 60 min. The selected snapshots in Figure 6B illustrate the dissolution of OSFs containing drug NCs over time. The area in blue represents the background (blank) while red colors indicate higher concentrations of dissolved drug (e.g., a deep red color corresponds to high concentration). The photographic examination of sample snapshots indicates an enhancement of color shade (light blue to dark red) with thickness–unit dose of OSFs as well as dissolution time.

From these recorded pictures, it is shown that different OSFs have different IDR profiles, which change as a function of the dissolution time and sample thickness–unit dose. The quantification of dissolved drug was selected at different times is shown in Supplementary Figure S4A. Surface drug concentration and sample mass released were proportional to the unit dose, which in turn was proportional to the thickness of the OSFs. Supplementary Figure S4B displays the IDR for OSFs, which were calculated using the known medium flow rate, measured concentration in the specified pixel area, and surface area of the sample [49,50,51,52,53]. Commonly, the highest absorbance profile for each OSF sample was obtained around 10 min to 60 min after a dissolution run started. By comparison, FNB-NC-E and the other three samples exhibited greater swelling, as indicated by their relatively thicker swelling bumps. FNB-NC-A had the highest dissolution rate, while FNB-NC-E and FNB-NC-C exhibited the highest IDR and surface concentrations. These values were comparable to the USP-IV dissolution data (Figure 6A). Finally, FNB-NCs from FNB-NC-E took longer to appear on sample surfaces before being released into release media for quantification by UV-absorption. Although the results obtained by using this methodology were equivalent to those of a more conventional dissolution method, the UV imaging setup may be applied for quantitative and qualitative analysis of dissolution patterns of BCS-II drugs.

### 3.7. Pharmacokinetic Parameters

**Figure 7:**
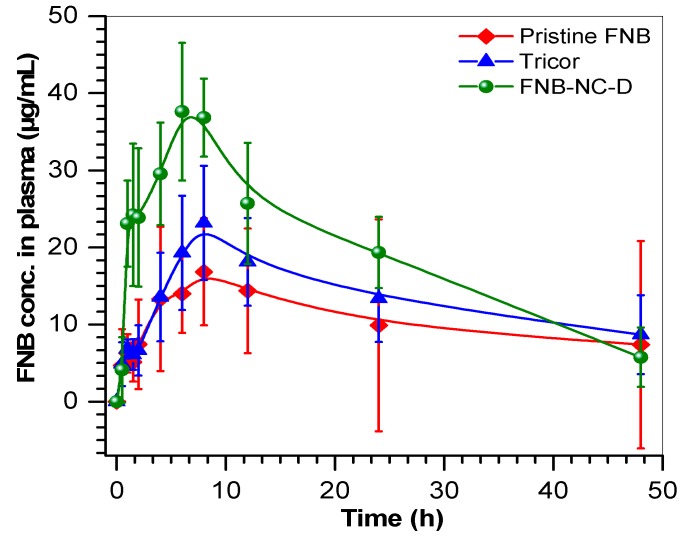
Time profiles of relative plasma concentrations of FNB after oral administration to rabbits when formulated in OSFs as compared to pristine FNB and Tricor; results are shown as means ± SD of six animals per group.

Pharmacokinetic study focused on investigating the feasibility of OSFs for enhanced bioavailability of FNB. The PK parameters, C_max_, T_max_ and AUC_0–∞_ and MRT were calculated from plasma drug concentration versus time profiles (Table 2 and Figure 7). PK data were shown higher C_max_ of OSFs (FNB-NC-D) (37.6 μg/mL) with respect to marketed Tricor formulation (23.18 μg/mL) and pristine FNB (16.84 μg/mL).The AUC_0–∞_, which represented the magnitude of drug absorption, was also significantly higher for OSFs formulation (931.26 μg·h/mL) compared to Tricor (654.6 ± 251 µg·h/mL) and pristine FNB (514.8 ± 374 µg·h/mL). The T_max_ (h) of OSFs was found to be 2 h earlier than the T_max_ of Tricor and pristine FNB. Also, OSFs showed reduction of the MRT (~4 h) compared to the both controls (Tricor and pristine FNB). These changes in T_max_ or MRT of the OSF formulation as compared to pristine FNB and Tricor are attributable to enhanced solubility of FNB nanocrystals redispersed *in vivo* from OSFs. Overall, PK study revealed that the nanocrystals formulated in OSFs showed better C_max_, T_max_, and AUC_0–∞_ than the marketed formulation (Tricor) and as received (pristine) FNB. OSFs formulation showed 1.4-fold enhancement in oral bioavailability when compared to controls. Due to the adhesive properties of OSFs, high surface area would increase influence by increased contact time with buccal mucosa. Accordingly, it seems that incorporation of drug nanocrystals into OSFs could highly improve oral bioavailability.

## 4. Conclusions

The main goal of this study was to develop fenofibrate (BCS-II, cholesterol reducing drug) nanocrystals and further mold into OSFs for enhanced oral bioavailability. To accomplish this aim, fenofibrate nanocrystals and OSFs were prepared by wet media milling technique and solvent free film casting method, respectively. However formulating of OSFs, HPMC/SDS ratio, the effect of films thickness and the particle size, zeta potential, drug content uniformity and redispersibility in aqueous medium were assessed. SEM image of fenofibrate nanocrystals and OSFs formulation shows the formation of nanocrystalline drug particles and uniform distribution in the OSFs, respectively. Solid-state characterizations suggested that the fenofibrate nanocrystals incorporated into the OSFs remained in the crystalline state and stable. Effect of OSFs thickness on *in vitro* drug release profile was tested in automated USP-IV. The release patterns were also tested *in situ* UV-dissolution imaging system. The out of five OSFs formulations, one of the best formulations (FNB-NC-D) was also examined for pharmacokinetics. A pharmacokinetics study was performed in rabbits by using suitable control groups. The pharmacokinetics result showed a significant difference in the pharmacokinetics parameter (1.4 to 1.8 fold increase in AUC_0–∞_). Thus, the developed OSFs demonstrated improvement of oral bioavailability. This platform is appropriate to BCS-II drugs and represents a promising approach to enhanced bioavailability.

## Figures and Tables

**Table 1:** Effect of thickness of fast dissolving oral thin films (OSFs) on drug loading.

Formulations	Film Thickness (µm)	wt FNB (%)	FNB Conc. (mg/cm^2^)
FNB-NC-A	45.90 ± 2.7	37.07 ± 1.4%	2.24
FNB-NC-B	84.5 ± 8.0	36.87 ± 0.9%	3.47
FNB-NC-C	115.8 ± 4.7	36.00 ± 0.7%	4.46
FNB-NC-D	151.2 ± 5.7	33.15 ± 0.3%	5.20
FNB-NC-E	165.0 ± 5.5	31.43 ± 1.5%	5.28

**Table 2:** Pharmacokinetic parameters of pristine FNB, Tricor, and OSFs in rabbits.

PK Parameters	FNB	Tricor	FNB-NC-D (OSF)	*p* Value
C_max_ (µg/mL)	16.8 ± 12.2	23.1 ± 8.8	37.6 ± 10.6 *	0.01
T_max_ (h)	8.0 ± 5.8	8.0 ± 3.0	6.0 ± 1.7	0.6
AUC _0–∞_ (µg·h/mL)	514.8 ± 374	654.6 ± 251	931.2 ± 263	0.08
MRT (h)	21.6 ± 15.7	21.4 ± 8.2	17.4 ± 4.9	0.7

*p* value is for one-way ANOVA. * *p* < 0.05 as compared to FNB group as per Tukey-Kramer multiple comparison test.

## Supplementary Materials

The following are available online at http://www.mdpi.com/2306-5354/5/1/16/s1, Figure S1: Average UV absorbance time profiles obtained by flowing drug standard solutions through the cell (*n* = 3). The absorbance values were obtained by averaging the pixels within the fixed areas (A) and the calibration curve constructed from plateau absorbance values. (Error bars represent the standard deviation (*n* = 3), and the solid line was obtained by linear regression analysis) (B); Figure S2: Schematic representation of the UV imaging setup (A) and OSFs sample mounting method (B); Figure S3: The FT-raman patterns; Figure S4: The surface concentration (A) and intrinsic dissolution rates (IDR) (B) of FNB-NCs from OSFs as a function of time, obtained by *in situ* real-time UV imaging at a flow rate of 100 μL/min; error bars represent the standard deviation (*n* = 3); Figure S5: Fitting the drug releases data to Higuchi (a), Korsmeyer–Peppas (b), parabolic diffusion (c), Elovich equation (d), Bhaskar-Equation (e), and Modified-Freundlich (f) kinetic models; Table S1: Mechanical strength analysis; Table S2: Linear Correlation Coefficient (r^2^) and rate constant (K) of the diffusion kinetic models applied to drug nanoparticles release from fast dissolving oral thin films (OTFs) (release data was consideration from first 24 min at pH 8.1).

Supplementary File 1
