# Supplementary material for: Fenofibrate Nanocrystals Embedded in Oral Strip-Films for Bioavailability Enhancement
Source: Bioengineering (Basel). 2018 Feb 13;5(1):16. doi: 10.3390/bioengineering5010016 (PMC5874882; doi:10.3390/bioengineering5010016)
Supplement: Supplementary File 1 [file bioengineering-05-00016-s001.docx]

**Supplementary materials**

**Method**

*Establishing calibration curve of drug in Surface Dissolution Imager*

The process for imaging of standards was in accordance with the previously described methodology [1-5]. Briefly, calibration curves for the UV imager were constructed using eight standard drug concentrations: 1.95 ×10^-5^, 2.93 ×10^-5^, 3.91 ×10^-5^, 5.86 ×10^-5^, 7.81 ×10^-5^, 1.17 ×10^-4^, 1.56 ×10^-4^ and 2.34 ×10^-4^ M in aqueous SDS solutions (7.2 mg/ml, pH = 8.1). Recording of UV images was implemented while each of the standard solutions was infused for a period of 4 min at a flow rate of 2 ml/min. The blank dissolution media were infused before and after the series of standard drug solutions for 4 min in order to detect baseline drift [1-3]. The infusion sequence for constructing the calibration curves (eight standard solutions and SDS as baseline blank) was repeated in triplicate. Conversion of pixel intensities into absorbance values was performed using the Actipix D100 software. Absorbance vs. time profiles were obtained as a result of such an infusion schedule, as described previously [1-5]. Average absorbance values obtained from each standard concentration were then used to build a standardized curve (Supplementary materials, Figure S1).


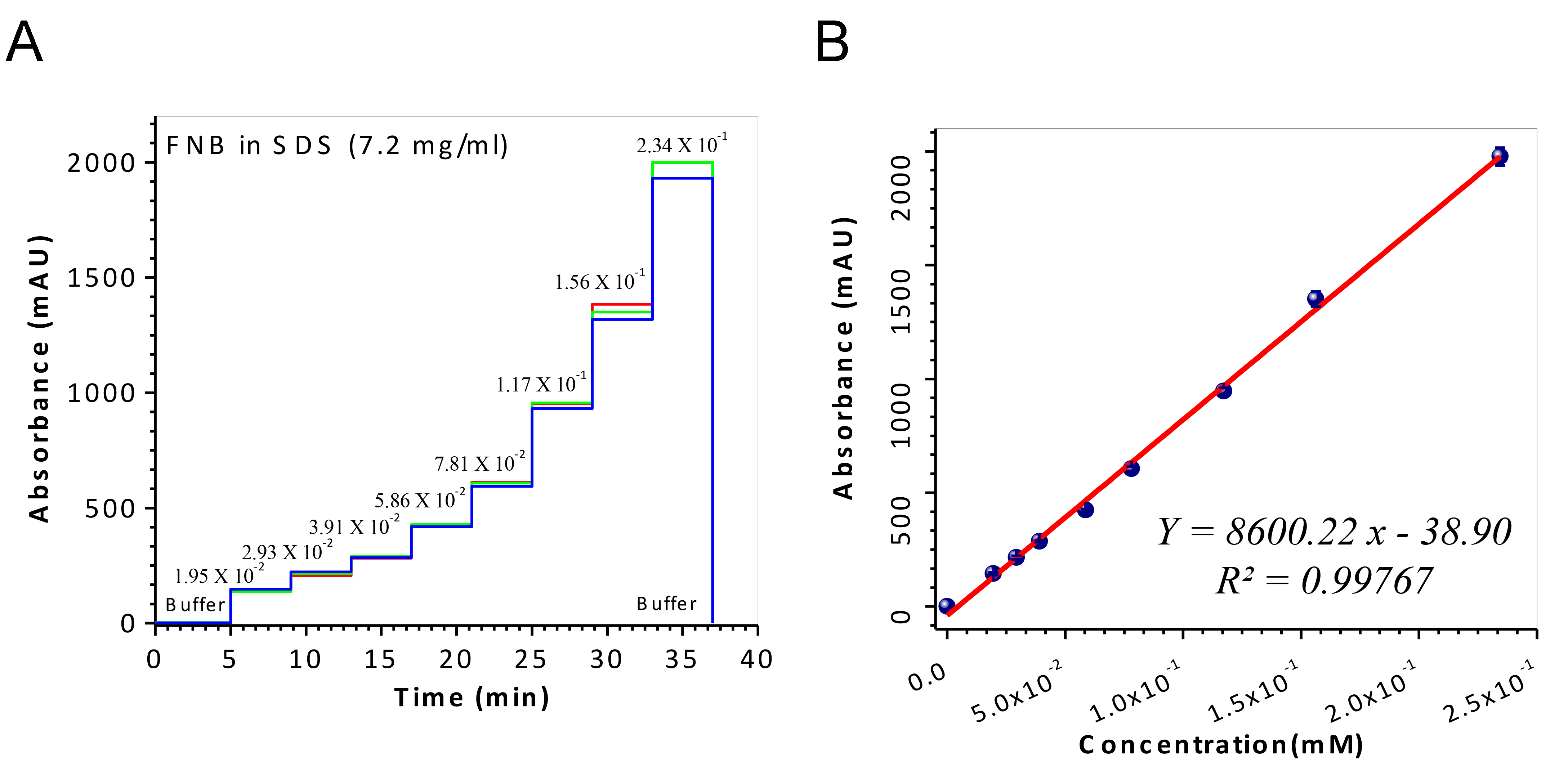


**Figure S1.** Average UV absorbance time proﬁles obtained by ﬂowing drug standard solutions through the cell (n = 3). The absorbance values were obtained by averaging the pixels within the fixed areas **(A)** and the calibration curve constructed from plateau absorbance values. (Error bars represent the standard deviation (n = 3), and the solid line was obtained by linear regression analysis) **(B)**.

**
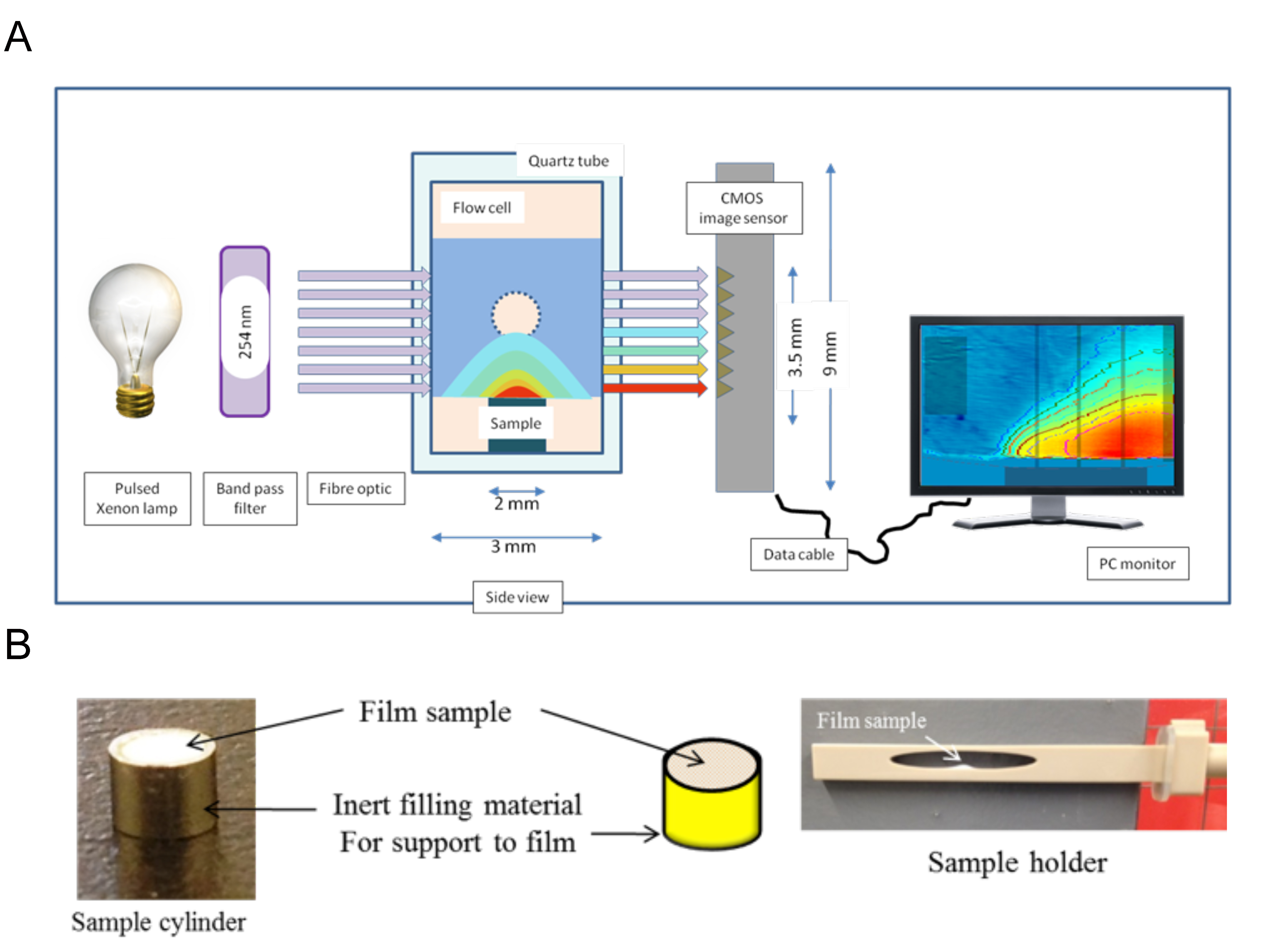
**

**Figure S2.**Schematic representation of the UV imaging setup **(A)** and OSFs sample mounting method **(B)**.

**Figure S3.** The FT-raman patterns

**
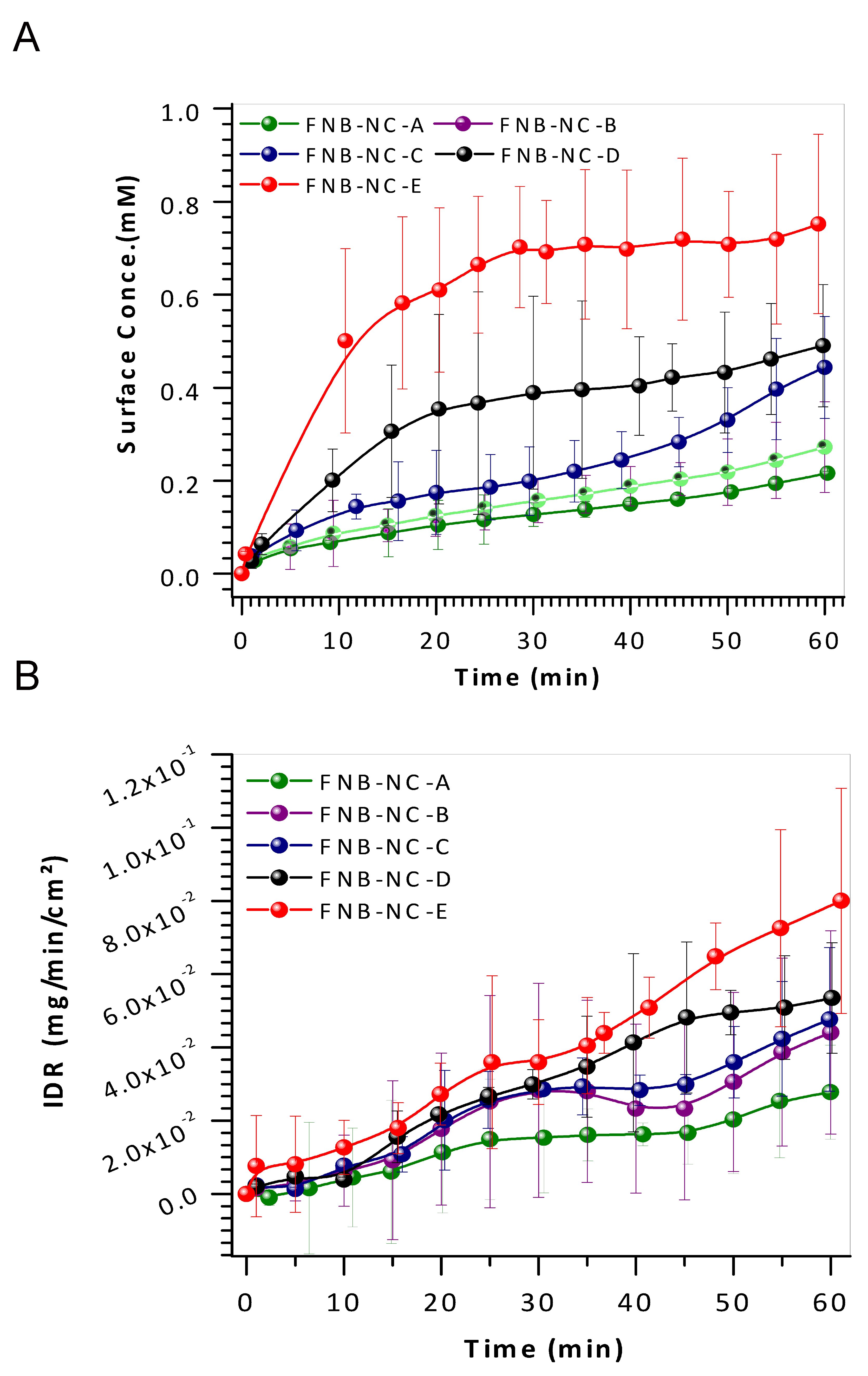
**

**Figure S4.** The surface concentration **(A)** and intrinsic dissolution rates (IDR) **(B)** of FNB-NCs from OSFs as a function of time, obtained by *in situ* real-time UV imaging at a ﬂow rate of 100 μL/min; error bars represent the standard deviation (n = 3).


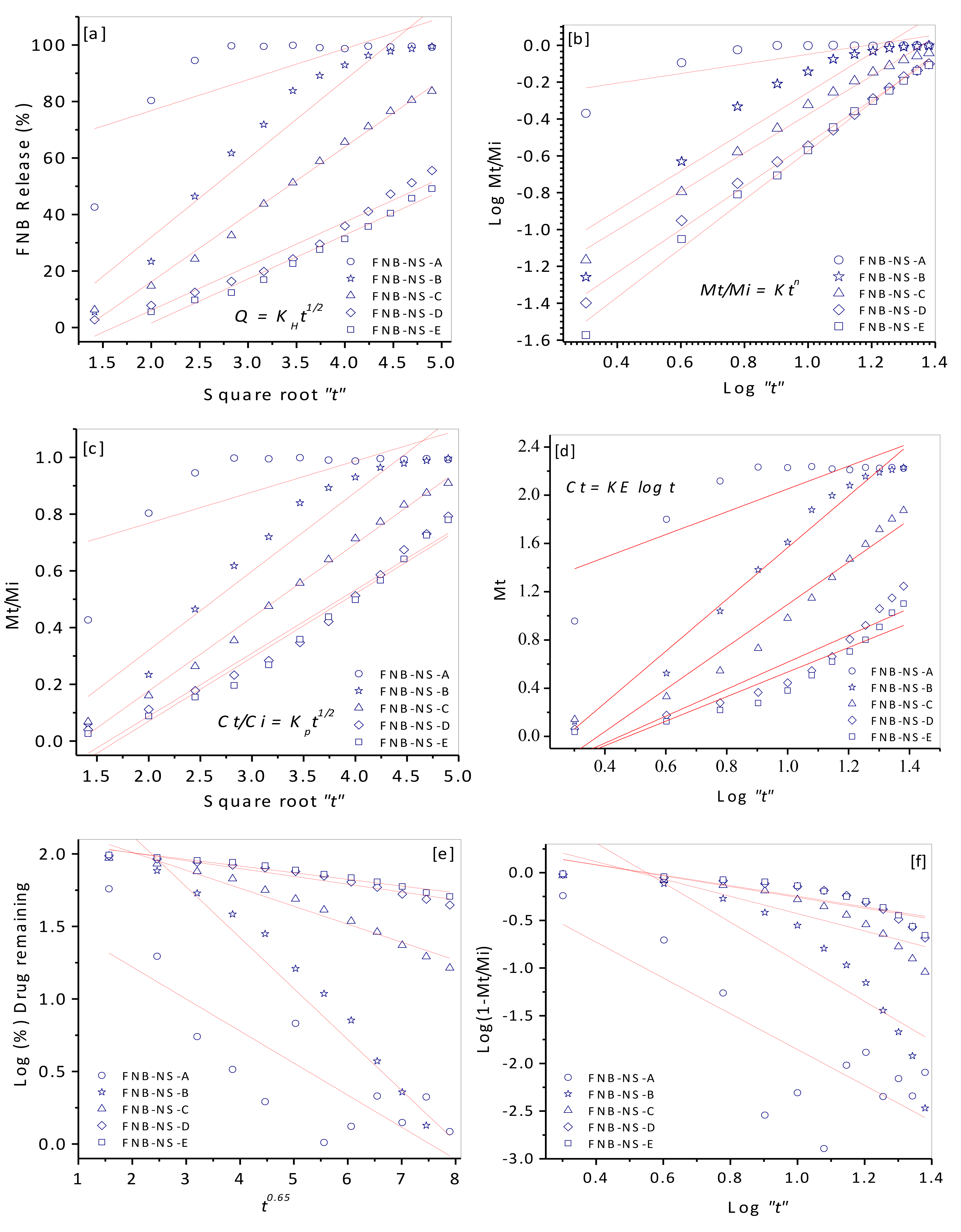


**Figure S5.**Fitting the drug releases data to Higuchi **(a)**, Korsmeyer–Peppas **(b),** parabolic diffusion **(c)**, Elovich equation **(d)**, Bhaskar-Equation **(e)**, and Modified-Freundlich **(f)** kinetic models.

*Drug release kinetics*

To understand the dissolution mechanism of drug NCs from the OSFs better, the Higuchi equation, Korsmeyer–Peppas equation, Elovich equation, Parabolic diffusion equation, Bhaskar equation, and Modiﬁed-Freundlich equation were fitted against the dissolution profiles presented in Fig. 7. Fitting results can be found in Fig.S5 (a–f) (Supplementary Materials, Table S3). The thicker OSFs (FNB-NC-C,D,E) were best fit by the Korsmeyer–Peppas model (*r^2^* = 0.986–0.995). The values of the correlation coefﬁcient (r^2^), release exponent (n) and rate constants (K_HP_) for the Korsmeyer–Peppas kinetic model are shown in Fig. S3b. Taking into account the value of n, FNB-NC dissolution from thinner OSF formulations (FNB-NC-A) followed the drug diffusion partially through a swollen matrix and water ﬁlled pores in the formulation (n < 0.5). Remaining formulations (FNB-NC-B, C, D, E) showed n > 1.0, suggesting super case II transport type of a release behavior. Thus, the kinetics of FNB dissolution was governed by erosion and swelling of HPMC matrix.

**Table S1.** Mechanical strength analysis

| Formulations | Tensile strength (MPa) | Yield strength (MPa) | Young's modulus (MPa) | Elongation at break (%) |
| --- | --- | --- | --- | --- |
| FNB-NS-A | 17.2±2.1 | 17.2±2.2 | 163.5±9.2 | 3.1±2.8 |
| FNB-NS-B | 17.8±0.4 | 15.1±0.8 | 186.3±5.8 | 4.3±0.7 |
| FNB-NS-C | 21.5±0.8 | 14.4±0.7 | 186.8±8.3 | 7.76±1.3 |
| FNB-NS-D | 26.1±3.2 | 18.3±2.0 | 221.0±2.0 | 10.1±2.5 |
| FNB-NS-E | 27.5±0.2 | 19.7±0.2 | 174.5±7.7 | 14.4±1.7 |

**Table S2.** Linear Correlation Coefficient (r^2^) and rate constant (K) of the diffusion kinetic models applied to drug nanoparticles release from fast dissolving oral thin films (OTFs) (release data was consideration from first 24 min at pH 8.1).

| Kinetic models | Parameters | Formulations | | | | |
| --- | --- | --- | --- | --- | --- | --- |
|  |  | **FNB-NC-A** | **FNB-NC-B** | **FNB-NC-C** | **FNB-NC-D** | **FNB-NC-E** |
| Higuchi-equation | *r^2^* | 0.4784 | 0.9248 | 0.9931 | 0.9561 | 0.9762 |
|  | *K_H_* | 10.95 | 27.80 | 23.86 | 15.60 | 15.58 |
| Korsmeyer-Peppas | *r^2^* | 0.6125 | 0.8679 | 0.9863 | 0.9950 | 0.9925 |
|  | *n* | 0.2608 | 1.0654 | 1.0450 | 1.1679 | 1.3209 |
|  | *K_HP_* | 0.4897 | 0.0476 | 0.0379 | 0.0199 | 0.0127 |
| Elovich-equation | *r^2^* | 0.6603 | 0.9768 | 0.9430 | 0.8547 | 0.8610 |
|  | *KE* | 0.9464 | 2.1479 | 1.7567 | 1.1181 | 1.0176 |
| Parabolic-diffusion | *r^2^* | 0.4784 | 0.9248 | 0.9931 | 0.9561 | 0.9612 |
|  | *Kp* | 0.1095 | 0.2784 | 0.2594 | 0.2225 | 0.2250 |
| Bhaskar-Equation | *r^2^* | 0.6705 | 0.9329 | 0.9597 | 0.9327 | 0.9472 |
|  | *K* | -0.2209 | -0.3512 | -0.1239 | -0.0540 | -0.0460 |
| Modified-Freundlich | *r^2^* | 0.5892 | 0.7588 | 0.7723 | 0.7068 | 0.7198 |
|  | *K* | -1.8769 | -2.0737 | -0.9073 | -0.5655 | -0.5535 |

**Reference**

1. Qiao, N.; Wang, K.; Schlindwein, W.; Davies, A.; Li, M. In situ monitoring of carbamazepine-nicotinamide cocrystal intrinsic dissolution behaviour. *Eur J Pharm Biopharm* **2013**, *83*, 415-426.

2. Boetker, J.P.; Savolainen, M.; Koradia, V.; Tian, F.; Rades, T.; Mullertz, A.; Cornett, C.; Rantanen, J.; Ostergaard, J. Insights into the early dissolution events of amlodipine using uv imaging and raman spectroscopy. *Mol Pharm* **2011**, *8*, 1372-1380.

3. Ostergaard, J.; Meng-Lund, E.; Larsen, S.W.; Larsen, C.; Petersson, K.; Lenke, J.; Jensen, H. Real-time uv imaging of nicotine release from transdermal patch. *Pharm Res* **2010**, *27*, 2614-2623.

4. Ostergaard, J.; Wu, J.X.; Naelapaa, K.; Boetker, J.P.; Jensen, H.; Rantanen, J. Simultaneous uv imaging and raman spectroscopy for the measurement of solvent-mediated phase transformations during dissolution testing. *J Pharm Sci* **2014**, *103*, 1149-1156.

5. Gordon, S.; Naelapaa, K.; Rantanen, J.; Selen, A.; Mullertz, A.; Ostergaard, J. Real-time dissolution behavior of furosemide in biorelevant media as determined by uv imaging. *Pharm Dev Technol* **2013**, *18*, 1407-1416.
